# Plasma Neurofilament Light Chain as a Predictive Biomarker for Post-stroke Cognitive Impairment: A Prospective Cohort Study

**DOI:** 10.3389/fnagi.2021.631738

**Published:** 2021-02-19

**Authors:** Zhiqiang Wang, Rongyu Wang, Yuxia Li, Mao Li, Yaodan Zhang, Lianyan Jiang, Jin Fan, Qingsong Wang, Dongdong Yang

**Affiliations:** ^1^Department of Neurology, Hospital of Chengdu University of Traditional Chinese Medicine, Chengdu, China; ^2^School of Clinical Medicine, Chengdu University of TCM, Chengdu, China; ^3^Department of Neurology, The General Hospital of Western Theater Command, Chengdu, China

**Keywords:** stroke, neurofilament light, biomarkers, post-stroke cognitive impairment, odds ratio, dementia

## Abstract

**Background:**

Plasma neurofilaments light chain (pNfL) is a marker of axonal injury. The purpose of this study was to examine the role of pNfL as a predictive biomarker for post-stroke cognitive impairment (PSCI).

**Methods:**

A prospective single-center observational cohort study was conducted at the General Hospital of Western Theater Command between July 1, 2017 and December 31, 2019. Consecutive patients ≥18 years with first-ever acute ischemic stroke (AIS) of anterior circulation within 24 h of symptom onset were included. PSCI was defined by the Montreal Cognitive Assessment (MOCA) (MOCA < 26) at 90 days after stroke onset.

**Results:**

A total of 1,694 patients [male, 893 (52.70%); median age, 64 (16) years] were enrolled in the cohort analysis, and 1,029 (60.70%) were diagnosed with PSCI. Patients with PSCI had significantly higher pNfL [median (IQR), 55.96 (36.13) vs. 35.73 (17.57) pg/ml; *P* < 0.001] than Non-PSCI. pNfL was valuable for the prediction of PSCI (OR 1.044, 95% CI 1.038–1.049, *P* < 0.001) after a logistic regression analysis, even after adjusting for conventional risk factors including age, sex, education level, NIHSS, TOAST classification, and infarction volume (OR 1.041, 95% CI 1.034–1.047, *P* < 0.001). The optimal cutoff value of the pNfL concentration was 46.12 pg/ml, which yielded a sensitivity of 71.0% and a specificity of 81.5%, with the area under the curve (AUC) at 0.785 (95% CI 0.762–0.808, *P* < 0.001).

**Conclusion:**

This prospective cohort study showed that the pNfL concentration within 48 h of onset was an independent risk factor for PSCI 90 days after an anterior circulation stroke, even after being adjusted for potential influencing factors regarded as clinically relevant.

**Clinical Trial Registration:**

www.chictr.org.cn, identifier ChiCTR1800020330.

## Introduction

Stroke survivors are at increased risk for cognitive impairment. Studies have shown that even mild strokes can increase the risk of cognitive impairment in survivors and affect their quality of life (Fride et al., 2015). Epidemiological studies have shown that stroke causes cognitive dysfunction in approximately one-third of patients, but it is unknown which stroke patients will suffer from cognitive impairment. Thus, the diagnosis and prediction of post-stroke cognitive impairment (PSCI) functional recovery by biomarkers has become a hot research topic. Previous studies have indicated that inflammatory biomarkers, growth factors, oxidative damage biomarkers, genetic biomarkers, and metabolic biomarkers in the circulating blood of patients may be the key determinants for the diagnosis and prediction of cognitive impairment (Zhang and Bi, 2020). However, these markers cannot reflect the mechanism of cognitive impairment caused by a stroke. The direct pathological cause of PSCI is neuronal damage in key brain regions, so looking for markers related to neuronal damage may predict and reflect a decline in the cognitive level.

Neurofilament light chain (NfL) is a neuron-specific structural protein (Zetterberg, 2016) that has recently been suggested as a marker of axonal injury and neurodegeneration with potential applications for both patient monitoring and for observational and interventional studies (Tiedt et al., 2018). In recent years, with the development of the quantitative detection technology of plasma NfL (pNfL), studies regarding the role of pNfL in neurodegenerative diseases and brain injury have been increasing (Gattringer et al., 2017; Guedes et al., 2020; Quiroz et al., 2020). Recent studies have observed a significant correlation among pNfL and the National Institutes of Health Stroke Scale (NIHSS) upon admission (Traenka et al., 2015; Al-Khaled, 2018), age-related white matter changes, infarct volume (Gattringer et al., 2017; Tiedt et al., 2018; Onatsu et al., 2019) and clinical outcome 90 days after stroke (Uphaus et al., 2019). A meta-analysis showed that the pNfL was a promising predictive biomarker for ischemic stroke outcome (Liu et al., 2020). The pNfL concentration had also been considered to be related to cognitive deterioration (Zetterberg et al., 2016; Mattsson et al., 2017; Olsson et al., 2019). To date and to our knowledge, the usefulness of pNfL for understanding PSCI is unclear. To address this, a prospective single-center observational cohort study was conducted to determine the association between pNfL and PSCI.

## Materials and Methods

### Study Design and Participants

This was a prospective single-center observational cohort study that included consecutive patients ≥18 years with first ever AIS of the anterior circulation within 24 h of symptom onset who were admitted to the General Hospital of Western Theater Command between July 1, 2017 and December 31, 2019. AIS was diagnosed according to the World Health Organization criteria and confirmed using brain computed tomography (CT) or magnetic resonance imaging (MRI). Patients were excluded if they (1) had a pre-existing cognitive impairment (clinical diagnosis or previous treatment or if the subject/caregiver reported progressive forgetfulness), mental illness or were unable to complete the cognitive assessments; (2) had issues combined with other non-vascular causes of neural function defects (brain injury, Alzheimer’s disease, Parkinson’s disease, and other neurological diseases);(3) were accompanied by serious medical diseases, tumor, hepatitis or an autoimmune disease; or (4) had survived less than 3 months.

Stroke severity was assessed using the National Institutes of Health Stroke Scale Score (NIHSS) and infarct volume (calculated using the MRI-DWI). The DWI lesion volumes were determined by the consensus of two experienced raters unaware of the clinical and laboratory results. The lesion size was calculated using the commonly used semiquantitative method (Broderick et al., 1993). All of the cases were invited for a 90-day follow-up visit. A physician blinded to the clinical data assessed changes in cognition. Post-stroke cognitive impairment (PSCI) was defined by the Montreal Cognitive Assessment (MOCA) (MOCA < 26) at 90 (± 5) days (Lees et al., 2014) after stroke onset via in-person interview.

### Blood Sampling and Biomarker Measurements

Venous blood samples were drawn upon admission or the next morning (within 48 h of stroke onset), and the time from stroke onset to blood collected was recorded. After centrifugation for 20 min at 3,000 g at room temperature, plasma (from the ethylene diamino tetraacetic acid (EDTA) tube) was aliquoted. The tubes were frozen locally at –80°C within 40 min after collection. The pNfL was measured using a single-molecule assay (SiMoA) platform (Quanterix, Lexington, MA, United States) as described (Weston et al., 2017; Tiedt et al., 2018). An in-house pool was used as an internal control and included in each assay for evaluating the assay performance. Samples were analyzed in duplicates, and the coefficient of variation (CV) was <11%.

### Statistical Analysis

Discrete variables were expressed as counts (percentages) and continuous variables as medians (interquartile range [IQR]). For the univariate analysis, the Mann-Whitney *U* test or chi-squared test was used to compare the demographic and clinical characteristics of patients with and without PSCI, as appropriate. The pNfL, MOCA, infarct volume, and NIHSS scores were log transformed (based 10) to closely normal distributions. In order to test for significant correlations between the clinical characteristics of patients and the plasma data, the Pearson correlational was used. The association of the pNfL levels upon admission with cognitive impairment was analyzed using a multiple logistic regression and adjusted for the established predictors. Variables that were identified as significant in univariate analyses (*P* < 0.1) were entered into the regression analyses together with other clinically significant variables. The optimal cutoff levels for the dichotomizing values were selected as the situation maximizing the Youden index. The receiver operating characteristic (ROC) analysis was performed to determine the sensitivity, specificity, and area-under-the ROC curve of the pNfL for detection of PSCI. In order to validate the model, 5-fold cross-validation was employed using the RMS package available on R statistical software. Other analyses were performed using SPSS 22 (IBM, Chicago, IL). All of the tests were 2-sided, and a *P* < 0.05 was considered to be significant.

## Results

A comparison of the demographic and clinical variables in the final dataset, including the pNfL data, is shown in Table 1. Of the 1,896 patients who were initially screened for eligibility, 202 (10.65%) were excluded for reasons that included: 68 (3.59%) death within 3 months, 45 (2.37%) combined with other central nervous system diseases, 43 (2.27%) unable to complete the cognitive assessments, 12 (0.63%) no plasma available, and 34 (1.79%) withdrew consent or were lost to follow-up. Finally, a total of 1,694 patients [male, 893 (52.72%); median age, 64 (IQR, 16) years] were enrolled in the cohort analysis, the median MOCA was 24 (IQR, 7), the median NIHSS was 4 (IQR, 7), the median blood sampling time was 19 h (IQR, 19; range 2–48), and the median pNfL was 46.41 (IQR, 36.26) pg/ml. A total of 1,029 (60.74%) were diagnosed with PSCI.

**TABLE 1:** Baseline characteristics of the participants.

Factors	Total	PSCI	Non-PSCI	*P*
Overall rate, *n* (%)	1,694(100)	1,029(60.74)	665 (39.26)	
Sex, male, *n* (%)	893 (52.72)	538 (52.28)	355 (53.38)	0.658
Age (y), median (IQR)	64.00 (16.00)	66.00 (18.50)	62.00 (13.00)	**<0.001**
Education level, <6 years, *n* (%)	900 (53.13)	561 (64.52)	339 (50.98)	0.154
BMI (kg/m^2^)	24.09 (1.53)	24.22 (1.59)	23.92 (1.43)	**<0.001**
**Vascular risk factors, *n* (%)**				
Hypertension	1,015(59.92)	630 (61.22)	385 (57.89)	0.172
Diabetes mellitus	566 (33.41)	346 (33.62)	220 (33.08)	0.817
Hyperlipidemia	385 (22.73)	249 (24.20)	136 (20.45)	0.072
Atrial fibrillation	371 (21.90)	267 (25.95)	104 (15.64)	**<0.001**
Smoking	478 (28.22)	276 (26.82)	202 (30.38)	0.113
Drinking	318 (18.77)	194 (18.85)	124 (18.65)	0.915
NIHSS, median (IQR)	4 (7)	6 (8)	3 (3)	**<0.001**
Infarct volume (ml), median (IQR)	15.83 (11.33)	15.99 (13.00)	15.10 (11.01)	**<0.001**
**TOAST classification, *n* (%)**				**<0.001**
Large-artery atherosclerosis	928 (54.78)	548 (53.26)	380 (57.14)	
Cardioembolism	239 (14.11)	175 (17.01)	64 (9.62)	
Small vessel occlusion	154 (9.09)	83 (8.07)	71 (10.68)	
Other cause	68 (4.01)	45 (4.37)	23 (3.46)	
Undetermined	305 (18.01)	178 (17.30)	127 (19.10)	
Blood sampling time (h), median (IQR)	19.00 (19.00)	20.00 (19.25)	17.00 (19.00)	0.471
pNfL (pg/mL), median (IQR)	46.41 (36.26)	55.96 (36.13)	35.73 (17.57)	**<0.001**
HbA1c (%), median (IQR)	5.9 (1.10)	5.9 (1.20)	5.9 (0.80)	0.901
HsCRP (mg/L), median (IQR)	3.58 (2.75)	3.51 (2.71)	3.61 (2.87)	0.332
HCY (μmol/L), median (IQR)	14.64 (9.45)	15.18 (9.24)	14.03 (10.15)	**<0.001**
MOCA, median (IQR)	24 (7)	21 (8)	27 (2)	**<0.001**

**PSCI, post-stroke cognitive impairment; IQR, interquartile range; BMI, Body Mass Index; pNfL, plasma neurofilament light chain concentration; NIHSS, National Institutes of Health Stroke Scale; and TOAST, Trial of ORG 10172 in Acute Stroke Treatment; HsCRP, high sensitivity C-reactive protein; HCY, homocysteine; MOCA, Montreal Cognitive Assessment. Bold text indicates a statistically less than 0.05.**

**FIGURE 1:**
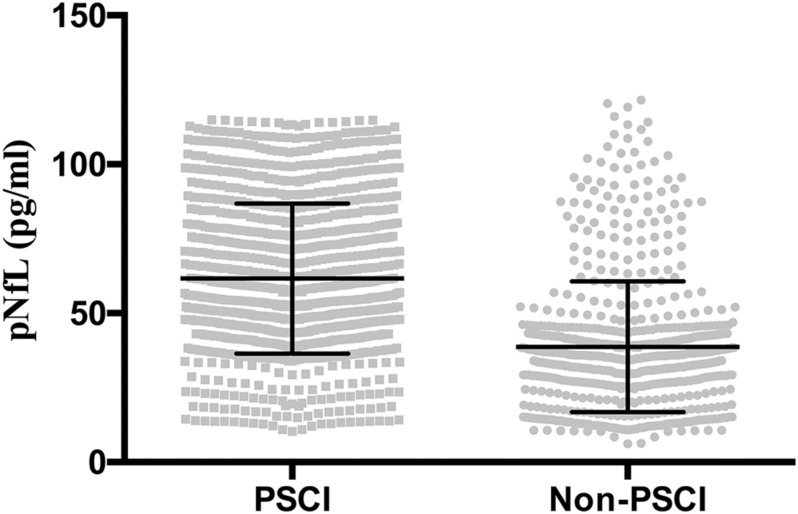
Plasma neurofilament light chain (pNfL) concentration in the diagnostic groups are shown as scatterplots. The pNfL concentration was higher in the post-stroke cognitive impairment (PSCI) group compared with the non-PSCI group (*P* < 0.001).

The PSCI and non-PSCI patients were well matched for sex, education level, history of hypertension, hyperlipidemia, diabetes, smoking, alcohol (all *P* > 0.05). However, the participants with atrial fibrillation, of older age, more clinical severity on the NIHSS, a larger infarction volume (ml), or a higher body mass index (BMI) were more common in the PSCI group (*P* < 0.05). No significant difference was observed between the two groups in terms of time to blood sampling (h), plasma high sensitivity C-reactive protein (HsCRP) level, and HbA1c levels (all *P* > 0.05). Compared with the non-PSCI group, the PSCI group exhibited significantly higher levels of pNFL and homocysteine (HCY) (both *P* < 0.05) (Table 1 and Figure 1).

**FIGURE 2:**
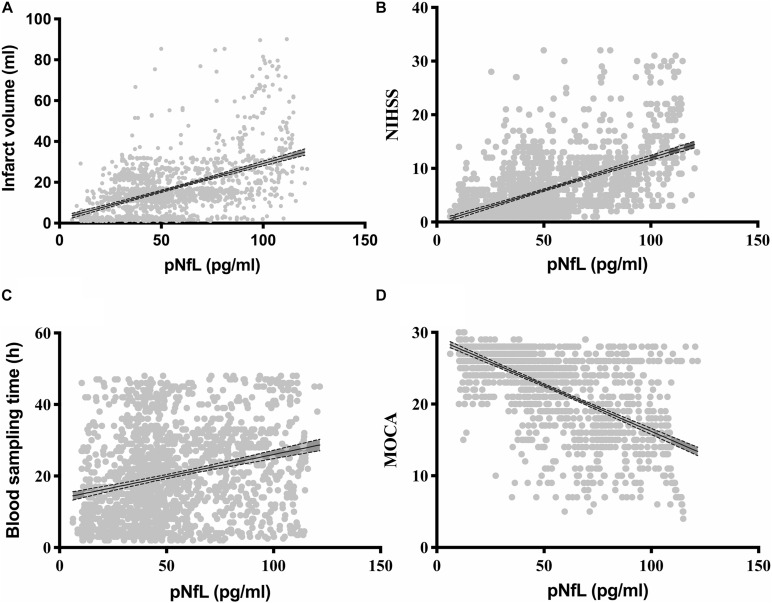
Correlations of the plasma neurofilament light chain (pNfL) concentration with **(A)** the infarction volume (ml), **(B)** the National Institutes of Health Stroke Scale (NIHSS), **(C)** the blood sampling time (h), and **(D)** the Montreal Cognitive Assessment (MOCA).

Correlation analysis showed that the log_10_ pNfL levels correlated with age (*r* = 0. 130, *P* < 0.001), log_10_ cerebral infarction volumes (*r* = 0. 509, *P* < 0.001; Figure 2A), the log_10_ NIHSS score (*r* = 0.510, *P* < 0.001; Figure 2B), and the log_10_ time to blood sampling (*r* = 0.261, *P* < 0.001; Figure 2C). The log_10_ pNfL levels were negatively correlated with cognitive impairment defined by log_10_ MOCA (*r* = −0.523, *P* < 0.001; Figure 2D).

A multivariable logistic regression revealed that patients with higher pNfL had a significantly higher risk of PSCI, even after adjusting for conventional risk factors including age, sex, education level, NIHSS score, TOAST classification and infarction volume (*P* < 0.05). This result indicated that the pNfL could be an independent risk factor for PSCI (Table 2).

**TABLE 2:** Logistic regression analysis for the association of pNfL with PSCI at 90-days.

Variables	OR	95%CI	*P*
Unadjusted pNfL	1.044	1.038–1.049	**<0.001**
Model 1 pNfL	1.044	1.038–1.050	**<0.001**
Model 2 pNfL	1.041	1.034–1.047	**<0.001**

**pNfL, plasma neurofilament light chain. Model 1 adjusted for age, sex, and education status. Model 2 adjusted for Model 1 and infarct volume, NIHSS and TOAST. Bold text indicates a statistical significance of less than 0.05.**

**FIGURE 3:**
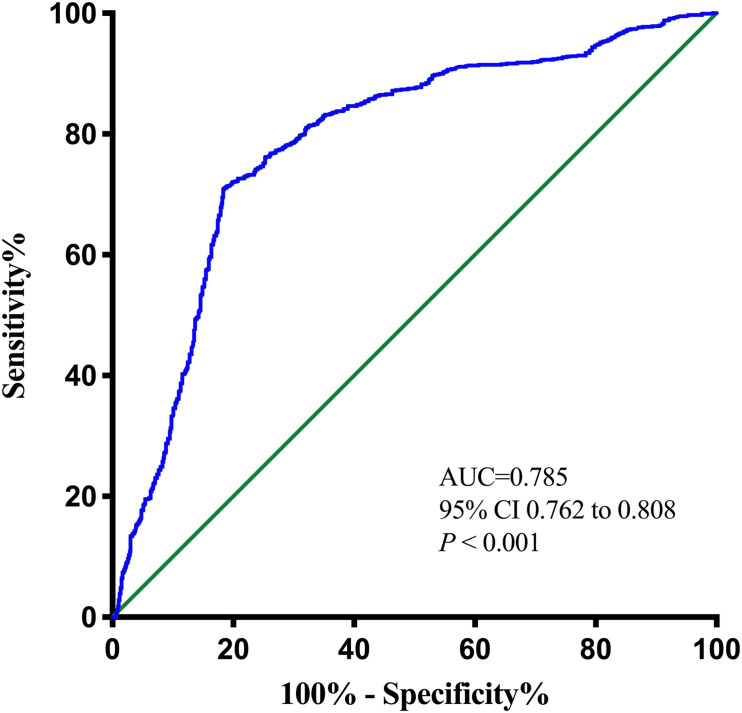
Receiver operating characteristic (ROC) curve for the plasma neurofilament light chain. AUC, area under curve.

The optimal cutoff value of the pNfL concentration as an indicator for auxiliary diagnosis of PSCI was assessed using the ROC curve. The optimal threshold was 46.12 pg/ml, which yielded a sensitivity of 71.0% and a specificity of 81.5%, with the AUC at 0.785 (95% CI, 0.762–0.808; *P* < 0.001; Figure 3). After 5-fold cross-validation, the c-statistic of the model was still 0.785. The calibration curve was very close to the actual curve, which showed that the model fits well.

## Discussion

This prospective cohort study showed that the pNfL concentration within 48 h of onset was an independent risk factor for PSCI within 90 days after an anterior circulation stroke, even after adjustment for potential influencing factors regarded as clinically relevant. In the present study, it was found that PSCI patients exhibited higher pNfL and Hcy levels than non-PSCI patients. Ages, BMI, atrial fibrillation, clinical severity on the NIHSS, and the infarction volume were also associated with cognitive function in post-stroke patients. The pNfL was positively correlated with the NIHSS score, infarct volume and the time to blood sampling time, negatively correlated with the MOCA. The levels of pNfL showed significant diagnostic accuracy in discriminating patients with PSCI from those without PSCI. This is the first study that has investigated the pNfL levels in patients with PSCI.

NfL, a neuron-specific structural protein (Zetterberg, 2016), has recently been suggested as a marker of neuroaxonal injury after ischemic stroke with potential application prospects for patient monitoring, observation, and intervention studies (Tiedt et al., 2018). Cerebrospinal fluid (CSF) NfL concentrations can be used as markers of axonal damage in white matter and other subcortical brain structures (Lycke et al., 1998; Zetterberg et al., 2006). Previous studies have shown that NfL expression was associated with dementia (Rosengren et al., 1999; de Jong et al., 2007; Howell et al., 2017; Zhao et al., 2019), small vessel disease (Gattringer et al., 2017), and other neurodegenerative diseases (Ge et al., 2018; Khalil et al., 2018; Bridel et al., 2019; Gagliardi et al., 2019; Gao et al., 2020). In addition, an increasing number of studies have demonstrated that pNfL levels were associated with clinical characteristics and outcome in stroke patients (Tiedt et al., 2018; Uphaus et al., 2019; Nielsen et al., 2020), and the CSF NfL increased months before the first dementia symptoms appeared, suggesting it might serve as a preclinical marker (Bacioglu et al., 2016). However, whether NfL expression is related to the occurrence of PSCI is still unknown. This study is the first to show that pNfL concentration was a blood marker of PSCI and has significant diagnostic accuracy in discriminating patients with PSCI from those without PSCI. The exclusion criteria of this study were not harsh; for example, patients with cortical infarction, large infarct size, or specific causes of stroke were not excluded. Hence, these findings could be considered representative of the spectrum of PSCI.

This study was the first to reveal the correlation between the NfL expression in plasma and the occurrence of PSCI. Previous studies have shown that the expression of NfL in the cerebral spinal fluid (CSF) was correlated with cognitive function, including Alzheimer’s disease (AD) (Weston et al., 2017), and frontotemporal dementia (FTD) (Rohrer et al., 2016), even in a small sample of vascular dementia (VaD) studies (Rosengren et al., 1999; Skillbäck et al., 2014). A network meta-analysis further demonstrated a significant increase in the CSF NfL expression level in dementias that engage the subcortical brain regions, such as VaD, than other types of dementia (Skillbäck et al., 2014; Zhao et al., 2019). Because the CSF collection is relatively complex, especially for stroke patients, the development of blood biomarkers (such as exosomes) is particularly important, and it is an important target for the future study of PSCI markers. The recent development of methods to quantify NfL in plasma had demonstrated that the pNfL concentration was closely correlated with cerebrospinal fluid NfL and directly reflected neurodegeneration within the central nervous system (Palermo et al., 2020). Consistent with what would be expected of a marker of neuronal damage, results from these studies showed a higher pNfL in patients with PSCI and correlations between pNfL and PSCI were observed (Gattringer et al., 2017; Tiedt et al., 2018). In agreement with earlier studies, it was found in this study that the severity of cognitive impairment increased with increasing pNfL levels (Gendron et al., 2020), making pNfL an easily accessible biomarker of the progression of neurodegenerative dementia diseases (Mattsson et al., 2017; Weston et al., 2017; Olsson et al., 2019).

In this study, a prospective cohort study was designed to show that pNfL was an independent risk factor for PSCI. Previous small-sample case-control studies only observed increased NfL expression in CSF of VaD patients (Sjögren et al., 2001; Wallin and Sjögren, 2001; Skillbäck et al., 2014; Zhao et al., 2019). In this study, by using a prospective cohort design, the regression analysis showed that pNfL expression was an independent risk factor for PSCI. The risks of PSCI were associated with age and vascular risk factors, such as atrial fibrillation, which was consistent with a previous study (Pendlebury et al., 2019). Also, stroke severity and infarction volume should be considered (Tiedt et al., 2018). In this study, all parameters were included when assessing the correlation of PSCI and pNfL. Nevertheless, after controlling for the confounders, the pNfL level displayed as an independent predictor of PSCI. The role of NfL in the pathophysiology of PSCI might be through some possible signaling pathways. pNfL is related to stroke severity (NIHSS score and lesion volume) and clinical outcomes (Onatsu et al., 2019; Pedersen et al., 2019), which are independent predictors of PSCI. However, pNfL still was a predictor for PSCI after adjusting for the NIHSS score and lesion volume, which was consistent with the results of a recent study (Gendron et al., 2020). The potential reasons behind why pNfL adds an additional predictive value apart from stroke severity should be considered. Acute infarcts further induce secondary neurodegeneration outside the infarct area, such as white matter tracts connected to the infarct. That secondary damage could contribute to poor cognitive and blocked neurotransmitter synthesis that leads to PSCI.

This study identified the correlation between pNfL and PSCI within 48 h of onset. There is evidence that pNfL increases with the time from symptom onset to blood-draw (Traenka et al., 2015; Gattringer et al., 2017; Tiedt et al., 2018), illustrating that the time point of measurement is of great importance when evaluating pNfL (Al-Khaled, 2018; Tiedt et al., 2018). To control the influence of blood collection time on the results, blood was collected within 48 h after the onset of the disease. It should be noted that patients within 24 h after stroke symptoms onset were included in this study. However, as some patients had their blood collected the next morning, the overall blood collection time was 48 h. This study showed that there was weak correlation between the concentration of pNfL and the blood collection time, and there was no difference in the blood collection time between groups. In contrast to a previous study that showed an association among functional outcomes 90 days after ischemic stroke and pNfL measured 7 days after symptom onset (Al-Khaled, 2018; Tiedt et al., 2018), as well as NfL that was independently correlated with the Mini-Mental State Examination at 0–8 days (Gendron et al., 2020), it was possible to already show a predictive effect of the NfL measured in plasma collected within 48 h after symptom onset. This was consistent with recent studies (Pedersen et al., 2019; Uphaus et al., 2019). This is especially important, as biomarker-based decision-making might be mandatory before the 7-day time point (Uphaus et al., 2019).

This study had several limitations. First, single-center cohorts, the exclusion of patients with aphasia or other severe conditions and patients in whom measurement of the pNfL levels failed may have led to an underestimation of the actual incidence of PSCI. Second, pNfL was measured only once. It may be essential to conduct a longitudinal study that measures at multiple time points after stroke to provide better prognostic information. Third, the centrifugal operation process was slightly different from the current standard guidelines (Tiedt et al., 2018), and the results should be further validated in future studies. Finally, single biomarkers may not be sufficient, and multiple biomarkers combined with a machine-learning algorithm should be used to automatically diagnose and predict PSCI.

## Conclusion

In conclusion, in this study, it was demonstrated that high pNfL levels within 48 h after first-ever anterior circulation stroke were associated with the development of PSCI 90 days after an acute ischemic stroke (AIS). In addition, this study showed significant diagnostic accuracy for discriminating patients with PSCI from patients without cognitive impairment. Further studies are needed to verify this association.

## Data Availability Statement

The raw data supporting the conclusions of this article will be made available by the authors, without undue reservation.

## Ethics Statement

The studies involving human participants were reviewed and approved by the Ethics Committee of General Hospital of Western Theater Command. The patients/participants provided their written informed consent to participate in this study.

## Author Contributions

DY: conceptualization, methodology, and software. QW: conceptualization, methodology, supervision, and investigation. ZW: data curation, writing the original draft, reviewing, and editing. RW: data curation and writing the original draft. ML and LJ: data curation. YL: data curation and investigation. YZ: software and validation. JF: supervision. All the authors reviewed the manuscript and approved the submitted version.

## Conflict of Interest

The authors declare that the research was conducted in the absence of any commercial or financial relationships that could be construed as a potential conflict of interest.
